# Short-term and long-term mate preference in men and women in an Iranian population

**DOI:** 10.1038/s41598-021-99653-7

**Published:** 2021-10-21

**Authors:** Fatemeh Sadat Mirfazeli, Meng-Chuan Lai, Amirhossein Memari, Armin Rajab, Milad Shafizadeh, Sahar Zarei, Seyed Vahid Shariat, Maryam Haghighi Fashi, Ebrahim Barzegary, Abdol-Hossein Vahabie

**Affiliations:** 1 Mental Health Research Center, Psychosocial Health Research Institute, Iran University of Medical Sciences, Tehran, Iran; 2 The Margaret and Wallace McCain Centre for Child, Youth & Family Mental Health and Azrieli Adult Neurodevelopmental Centre, Campbell Family Mental Health Research Institute, Centre for Addiction and Mental Health, Toronto, ON Canada; 3 Department of Psychiatry and Autism Research Unit, The Hospital for Sick Children, Toronto, ON Canada; 4 Department of Psychiatry, Temerty Faculty of Medicine, University of Toronto, Toronto, ON Canada; 5 Autism Research Centre, Department of Psychiatry, University of Cambridge, Cambridge, UK; 6 Department of Psychiatry, National Taiwan University Hospital and College of Medicine, Taipei, Taiwan; 7 Sports Medicine Research Center, Neuroscience Institute, Tehran University of Medical Sciences, Tehran, Iran; 8 Endocrinology and Metabolism Research Center (EMRC), Vali-Asr Hospital, Tehran University of Medical Sciences, Tehran, Iran; 9 Department of Neurosurgery, Tehran University of Medical Sciences, Tehran, Iran; 10 Department of Cognition and Communication, University of Copenhagen, Copenhagen, Denmark; 11 School of Behavioral Sciences and Mental Health (Tehran Institute of Psychiatry), Iran University of Medical Sciences, Tehran, Iran; 12 Los Angeles Community College, Los Angeles, USA; 13 Los Angeles City College, Los Angeles, USA; 14 Department of Marketing, University of Washington, Seattle, WA USA; 15 Cognitive Systems Laboratory, Control and Intelligent Processing Center of Excellence (CIPCE), School of Electrical and Computer Engineering, College of Engineering, University of Tehran, Tehran, Iran; 16 Department of Psychology, Faculty of Psychology and Education, University of Tehran, Tehran, Iran; 17 School of Cognitive Sciences, Institute for Research in Fundamental Sciences (IPM), Tehran, Iran

**Keywords:** Neuroscience, Cognitive neuroscience, Decision

## Abstract

Mate preference in short-term relationships and long-term ones may depend on many physical, psychological, and socio-cultural factors. In this study, 178 students (81 females) in sports and 153 engineering students (64 females) answered the systemizing quotient (SQ) and empathizing quotient (EQ) questionnaires and had their digit ratio measured. They rated their preferred mate on 12 black-line drawing body figures varying in body mass index (BMI) and waist to hip ratio (WHR) for short-term and long-term relationships. Men relative to women preferred lower WHR and BMI for mate selection for both short-term and long-term relationships. BMI and WHR preference in men is independent of each other, but has a negative correlation in women. For men, digit ratio was inversely associated with BMI (p = 0.039, B = − 0.154) preference in a short-term relationship, and EQ was inversely associated with WHR preference in a long-term relationship (p = 0.045, B = − 0.164). Furthermore, men and women in sports, compared to engineering students, preferred higher (p = 0.009, B = 0.201) and lower BMI (p = 0.034, B = − 0.182) for short-term relationships, respectively. Women were more consistent in their preferences for short-term and long-term relationships relative to men. Both biological factors and social/experiential factors contribute to mate preferences in men while in women, mostly social/experiential factors contribute to them.

## Introduction

Based on evolutionary theories, mate selection has been an adaptive challenge since the prehistoric period. Mate value is reliably signaled by physical attractiveness^1,2^. Waist to hip ratio (WHR) and weight (represented by body mass index, BMI) have been highlighted in the literature as cues for attractiveness cross-culturally^3–5^. Several studies have discussed the evolutionary advantage of these physical attractiveness cues. For example, the degree of fat storage (reflected by BMI) and its distributional pattern (reflected by WHR) may signal the health status and reproductive potential of the selected mate^6–9^. Body fat storage is an indicator for fertility, pregnancy, and lactation^10^. Moreover, body fat distribution is linked to the sex hormone status of individuals. During puberty, estrogen induces fat deposition in thighs, hips, and buttocks, leading to lower WHR, while testosterone stimulates fat storage in the abdomen, resulting in higher WHR^11–13^. Lower WHR (i.e., 0.7 or 0.8) reliably signals cardiovascular health and fertility in women^14–20^, while higher WHR (0.9) is considered healthy for men^19,21^. However, despite some cross-cultural cues for physical attractiveness^22^, recent research has indicated that individual differences in psychological traits can also lead to variations in mate preference^23–27^. For instance, Smith et al. argued that empathizing-systemizing “cognitive style” could affect our judgment of beauty^26^. In their study, heterosexual women with higher empathizing scores preferred mostly more masculine men (e.g., with robust jaw), and heterosexual men with higher systemizing scores were attracted mostly to women with more feminine features (e.g., with large eyes). Empathizing is a cognitive style associated with identifying the emotional state of other people, whereas systemizing is a cognitive style related to the tendency in analyzing and predicting rules and behaviors of rule-based systems. Large-scale studies repeatedly show that on average, women tend to score higher on empathizing tendencies while men tend to score higher on systemizing tendencies, and in particular, there are on-average differences between men and women on the discrepancy between empathizing and systemizing. These differences can be partly explained by the combination of social-cultural influences such as gender stereotypes^28^, varied brain structure^29^, and brain exposure to sex steroid hormones in the fetal period^30–34^. Accordingly, one could speculate that sex-typical psychological traits of systemizing and empathizing potentially influence the judgment of attractiveness in adults^26^. However, in this research direction, only face stimuli have been used so far as a representative of attractiveness, and there is a paucity of research examining the associations between psychological traits and other physical cues of attractiveness such as WHR and BMI, and we still do not know whether there is a correlation between a masculine cognitive style and a feminine women preference, or these cognitive style could provide more potentials for mate preferences.

In addition to the impact of sex-typical psychological traits on attractiveness assessment, mate preference in short-term versus long-term relationships are largely gender-dependent^35^. Mate selection in men seems to be different from women under short-term versus long-term conditions^36,37^. For example, Confer et al. showed that men but not women would give higher priority to facial cues for a long-term relationship while they would switch their priority to bodily cues for short-term mate selection^35^. Short-term and long-term relationships may differ as both men and women seek a qualified mate for having children in long-term relationships. It seems that however, men largely prioritize reproductive value in their female partner while women often prioritize resource acquisition ability in their male partner^37^. Women’s mate strategies might be less variable than men's^38^, in a way that women prefer similar characteristics for short-term and long-term relationships^39^. One reason would be short-term relationship would provide an assessment device for the women to investigate long-term prospects of their possible future mate with better genes^40^. Despite all these differences, it seems that men and women both value physical attractiveness, economic status, and fertility differently for a long-term relationship^41^. Men and women are both highly variable on their mate preference strategies and have evolved to be dependent on contextual effects and individual differences^42^.

Besides individual differences in sex-typical psychological traits, variation in second-to-forth digit ratio (2D:4D) has been suggested as a prenatally, biologically determined, sex-typical marker, which can influence mate preference^43,44^. Similar to empathizing-systemizing cognitive style, the 2D:4D digit ratio might be also influenced by fetal androgen exposure (i.e. inversely correlated with fetal androgen exposure)^45,46^. However, its role as a marker for mating preference (e.g., assortative mating) is still controversial^47^. Along with the paucity of research on the associations between cognitive style, digit ratio, and mate selection, the effects of other individual differences such as educational background are insufficiently explored. For example, most studies have focused on similarities in the educational background between couples^48^. Furthermore, the role of individual differences like empathizing-systemizing cognitive style as well as that of contextual effects in gender-related mate preference in short-term versus long-term relationships is also underexplored.

To address these knowledge gaps, we aimed to compare mate preference in men and women with two different educational backgrounds (i.e. sports/physical education versus engineering), one known for their masculine physical characteristics and the other for their masculine cognitive style^49^. We further examined the impact of cognitive style and digit ratio, two sex-typical characteristics, on WHR and BMI preferences in men and women. In addition, we compared mate preference in both short-term and long-term relationships. We hypothesized that our participants would generally follow stereotypical standards of attractiveness shown in their mate preference. Individual differences and exposure to specific environments such as sports environments would lead to within-population differences in mate preference, mostly in terms of short-term mating. We also hypothesized that exposure to a cultural environment with women with full-body coverage in society would underestimate the role of physical body figures in male mate preference and highlight the role of other physical characteristics and socio-cultural factors.

## Results

### Mate preference

The mean and standard deviation of mate preference (indexed by BMI and WHR) for short-term and long-term relationships for both male and female participants are described in Table 1.

**Table 1:** The mean value of BMI and WHR for short-term relationships and long-term relationships.

Male participants, short-term relationships		Male participants, long-term relationships		Female participants, short-term relationships		Female participants, long-term relationships	
BMI (mean ± SE; skewness)	WHR (mean ± SE; skewness)	BMI (mean ± SE; skewness)	WHR (mean ± SE; skewness)	BMI (mean ± SE; skewness)	WHR (mean ± SE; skewness)	BMI (mean ± SE; skewness)	WHR (mean ± SE; skewness)
− 0.4754 ± 0.0444; 0.6685	0.8000 ± 0.0007; 0.2818	− 0.4863 ± 0.0423; 0.5622	0.7978 ± 0.0007; 0.5706	0.0347 ± 0.0634; − 0.0578	0.8639 ± 0.0008; 0.0189	0.0069 ± 0.0603; − 0.0104	0.8667 ± 0.0008; − 0.0938

**Figure 1:**
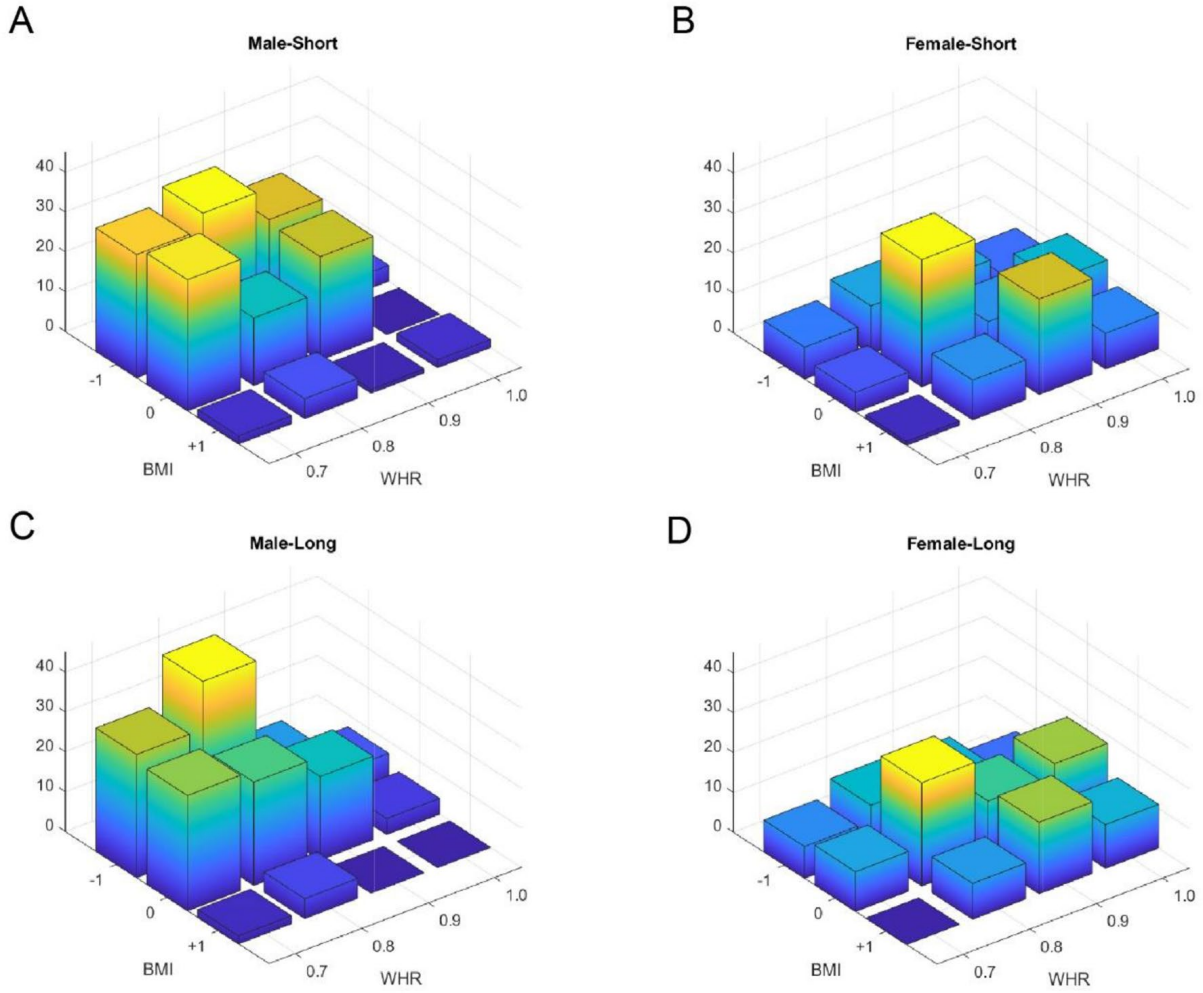
Histogram of the most attractive selected stimuli. X and y axes show BMI and WHR respectively, and the z-axis shows the number of participants that have selected the corresponding bin of the histogram. (**A**) Male-Short: male participant selecting for a short-term relationship; (**C**) Male-Long: male participant selecting for a long-term relationship; (**B**) Female-Short: female participant selecting for a short-term relationship; (**D**) Female-Long: female participant selecting for a long-term relationship.

Figure 1 descriptively illustrates the distribution of the most preferred mate for short-term and long-term relationships for male and female participants separately. Male participants preferred female figures with normal and underweight BMI and lower WHR in both short-term and long-term relationships; female participants preferred male figures with normal BMI and higher WHR. Females’ mate preference showed extended distribution towards both underweight and overweight ends and so the BMI distribution for females had higher variance relative to males’ BMI distribution (two-sample *F*-test of equality of variances between males and females’ BMI): for short-term relationships (females SD: 0.761; males SD: 0.601; F(143,182) = 1.606, p = 0.0026) and for long-term relationships (females SD: 0.724; males SD: 0.573; F(143,182) = 1.598, p = 0.0028). The same analyses on WHR do not show any significant difference between males and females (two-sample *F*-test of equality of variances between males and females’ WHR): for short-term relationships (females SD: 0.921; males SD: 0.890; F(143,182) = 1.071, p = 0.658) and for long-term relationships (females SD: 0.982; males SD: 0.883; F(143,182) = 1.238, p = 0.175). Moreover, distributions of BMI and WHR in females were more symmetric (has lower skewness; see Table 1) relative to those of males, that had higher skewness.

There was no significant correlation between WHR and BMI for male participants in mate preference for short-term relationships (r = − 0.01, p = 0.89) or in long-term relationships (r = − 0.02, p = 0.77). There was a significant correlation between BMI and WHR for female participants in mate preference for short-term relationships (r = 0.20, p = 0.02) and long-term relationships (r = 0.24, p = 0.003). Fisher’s r-to-z test for difference between correlations in male and female participants showed significant differences in a short-term relationship (z = 1.97, p = 0.05) and long-term relationship (z = 2.36, p = 0.019). Women preferred higher WHR in higher BMI figures. This indicated a gender-differential mate preference pattern, that men chose the most attractive WHR and BMI independently, while there was a small but significant positive correlation between the two for women.

### Regression-based analysis of mate preference

To determine the factors that predict mate preferences, we conducted regression analyses using the following factors: education background group (sports vs. engineering), SQ, EQ, digit ratio, and age. The regression analyses were done separately for males and females, and also for short-term and long-term relationships. Coefficients were reported as normalized by their standard error and so they were t-statistics of regression coefficients. Normalized coefficients and p-values for each analysis were shown in Table 2.

**Table 2:** Results of regression analysis for prediction of preferred WHR and BMI for short-term and long-term relationships, separately in female and male participants.

	Educaional background	SQ	EQ	Digit ratio	Age
**Males’ short-term preference **					
BMI					
Beta	0.201	− 0.018	− 0.044	− 0.154	− 0.016
p-value	0.009*	0.822	0.586	0.039*	0.833
WHR					
Beta	− 0.086	0.032	0.074	− 0.063	− 0.136
p-value	0.263	0.687	0.365	0.40	0.081
**Males’ long-term preference**					
BMI					
Beta	0.131	0.0050	− 0.067	− 0.060	− 0.085
p-value	0.092	0.949	0.415	0.43	0.28
WHR					
Beta	− 0.050	0.123	− 0.164	0.072	− 0.052
p-value	0.518	0.122	0.045*	0.339	0.504
**Females’ short-term preference**					
BMI					
Beta	− 0.182	− 0.0090	− 0.020	− 0.154	0.078
p-value	0.034*	0.917	0.82	0.070	0.355
WHR					
Beta	− 0.124	− 0.142	0.029	− 0.067	− 0.091
p-value	0.152	0.114	0.743	0.434	0.285
**Females’ long-term preference **					
BMI					
Beta	− 0.073	0.0080	0.077	− 0.114	0.152
p-value	0.40	0.931	0.385	0.184	0.077
WHR					
Beta	0.0030	− 0.060	0.0080	− 0.0050	− 0.074
p-value	0.974	0.508	0.929	0.954	0.398

*p-value < 0.05 is considered significant.

There was a significant difference (z-test between regression coefficients: z = 3.34, p = 8e−4) in the most attractive BMI for male and female participants for short-term relationships, in the opposite directions; i.e. males in sports preferred higher BMI (i.e. positive beta), while females in engineering education preferred higher BMI (i.e. negative beta). Men with higher digit ratio preferred underweight BMI and men with lower digit ratio mostly preferred normal BMI. This relationship did not hold for long-term relationships in male participants. EQ was related to mating preference based on WHR in long-term relationships in male participants, that men with higher EQ preferred lower WHR. These regression-based results should be considered suggestive rather than confirmative, as they have not been corrected for multiple comparisons.

### Mate preference in short- vs. long-term relationships

**Figure 2:**
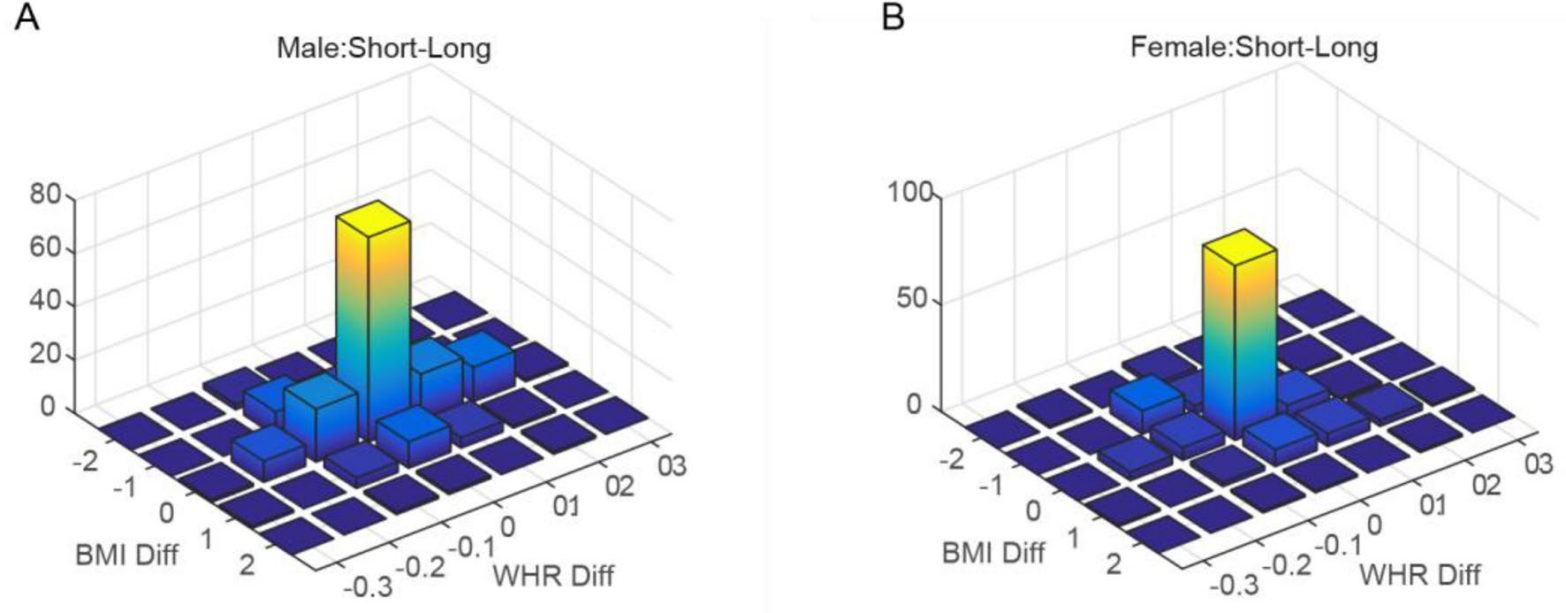
Histograms of differences of BMI and WHR between short-term and long-term relationships for male participants (**A**) and female participants (**B**). BMI Diff and WHR Diff in x and y axes indicate the selected BMI and WHR in a short-term relationship minus the same in a long-term relationship for each participant.

The next question regards how many men and women are consistent in their mate preferences in short-term and long-term relationships, and whether they select a similar mate for short-term and long-term relationships. The main hypothesis was that women put more emphasis on long-term relationships and their short-term and long-term mate preferences would be more consistent, while men might prefer different mate preferences for short-term and long-term relationships. Figure 2 descriptively illustrates the distribution of the difference (in BMI or WHR) between the preferred stimuli, in short-term minus long-term relationships for male and female participants, separately. These difference distributions show that how much mate preference based on BMI or WHR is preserved across short-term and long-term relationships. It showed that 42% of men and 57% of women did not show different mate preferences for short-term vs. long-term relationships, i.e. they chose the same BMI and WHR as the most attractive figure for short-term and long-term relationships. Investigation of the percent of participants who did not differ in their short-term and long-term relationship mate preferences (in BMI or WHR) implied that 75% of men and 69% of women selected the same BMI both for their short-term and long-term relationships, while 50% of men and 67% of women prefer the same WHR for their relationships.

To assess whether the consistency between short-term and long-term relationships are significantly different between men and women, we compared the correlation coefficient between long-term and short-term preferences in men and women. The correlation coefficient between long-term and short-term BMI for men (r = 0.522, p = 3.4e−14) was not significantly different (z = 0.769, p = 0.442) than that for women (r = 0.609, p = 5.9e−16). The correlation coefficient between long-term and short-term WHR for men (r = 0.349, p = 1.2e−6) was significantly lower (z = 2.504, p = 0.012) than that for women (r = 0.631, p = 2.2e−17).

To examine whether changes in short-term vs. long-term relationships are interrelated between BMI and WHR (i.e. if the preferred BMI or WHR depends on the other factor), we analyzed the difference of BMI in short-term and long-term relationships with the difference of WHR in short-term and long-term relationships. There was no significant correlation between the changes in BMI and the changes in WHR in the short-term relative to the long-term relationship for men (r = 0.066, p = 0.38) while there was a significant correlation for women (r = 0.31, p = 0.0001). The correlation coefficient in women was significantly different from men (Fisher’s r-to-z-test, z = 3.42, p = 6e−4). This shows that the correlation between BMI and WHR in the previous section is extended into the variation of preferences for short-term and long-term relationships in women. These correlations suggest that the mate preference based on either aspect (BMI or WHR) was interrelated in the eyes of women, while in men the preference based on BMI or WHR was independent. Table 3 illustrates a regression analysis of these differences as dependent variables with the same covariates as previous regression analyzes. We found that the EQ score was related to the change of WHR preference between short-term and long-term relationships in men.

**Table 3:** Results of regression analysis for prediction of difference of preferred WHR/BMI in short-term relationship minus the preferred WHR/BMI in long-term relationship, separately in female and male participants.

	Educaional background	Systemizing Quotient (SQ)	Empathizing Quotient (EQ)	Digit ratio	Age
**Males’ short-term relationship minus long-term relationship**					
BMI					
Beta	0.079	− 0.024	0.021	− 0.102	0.067
p-value	0.31	0.769	0.800	0.18	0.391
WHR					
Beta	− 0.033	− 0.079	0.208	− 0.118	− 0.074
p-value	0.671	0.316	0.011*	0.115	0.338
**Females’ short-term relationship minus long-term relationship**					
BMI					
Beta	− 0.13	− 0.019	− 0.107	− 0.053	− 0.077
p-value	0.131	0.83	0.223	0.537	0.368
WHR					
Beta	− 0.143	− 0.087	0.023	− 0.069	− 0.014
p-value	0.102	0.335	0.797	0.422	0.869

*p-value < 0.05 is considered significant.

## Discussion

The present research provides the first evidence that preference for specific body features is linked to indirect indices of the fetal hormonal environment (e.g., digit ratio and empathizing-systemizing cognitive style), and such relationships differ in men and women. Our study extends previous research on the association between cognitive style and face preference^26^. In the current study, male participants generally complied with stereotypical norms of attractiveness (i.e. preference for low WHR and normal-to-low BMI for men in both short-term and long-term relationship contexts). Finding a connection between low WHR and attractiveness in the Iranian population, in a non-western country, reinforces the assumption that there are potential culturally invariant factors in mate preferences^14,21,50^. Individual differences such as lower digit ratio in men and educational background in both men and women in a short-term relationship are associated with the preference of stereotypical standard BMI. Accordingly, in long-term relationships, the higher empathizing tendency in men increased their preference for lower WHR, which again is a stereotypical beauty norm. We also found that women were different from men in mate preference over different individual and contextual factors (e.g., duration of relationship).

We found that in men, a lower digit ratio was associated with a preference for higher BMI (which in the present study reflects a tendency towards a normal and underweight BMI see Fig. 1) for a short-term relationship. This implies that fetal hormonal exposure may reinforce men’s mate preference towards existing norms. Another determining factor of short-term mate preference was the educational background. Men in sports compared to men in engineering also preferred higher BMI (which in the present study reflects a tendency towards a normal and underweight BMI see Fig. 1) for a short-term relationship. It appears that men with prominent physical sex-stereotypical characteristics, i.e. lower digit ratio^46^ and sports background, follow common stereotypical beauty trends based on BMI. Individuals of high mate value that is men with prominent physical sex-stereotypical characteristics would be more able to follow common stereotypical physical attractiveness^40^.

The selection of low WHR as a fertility predictor of the mate^15,16^ was even reinforced in men with a higher empathizing tendency in a long-term relationship. From an evolutionary perspective, empathizing tendency may facilitate men to be attracted to women with lower WHR or facilitate skills which is necessary for persuading a fitter mate with lower WHR for their long-term relationship, in which one may be more fertile and more probable to have offspring. Furthermore, empathizing tendency might come with better parental skills and higher socializing capacity with offspring in long-term relationship in which one prioritizes productivity^40,^ therefore the preference of lower WHR preference, a marker of beauty, fertility and productivity, is reinforced ^18,51^.

Observing low WHR preference in men with high empathizing cognitive style is almost in line with findings from Brosnan and Walker^52^, who reported that fathers of children with autism (i.e. with higher systemizing capabilities or lower empathizing capabilities) preferred women with higher-than-average WHR, reflecting male-like patterns of WHR (i.e. high WHR may tract with higher current testosterone^53^). Higher testosterone in the prenatal environment is associated with a higher probability of offspring with autism^54,55^. Therefore, selecting a female partner with higher WHR may increase the likelihood of having a child with autism^52^. A high empathizing cognitive style may be protective against selecting a partner with a high testosterone level, in which case there is a higher possibility of having an offspring with autism^56,57^. Our finding of low WHR preference (i.e. feminine body figure) in men with high empathizing tendency, however, is somehow in contrast with the findings of Smith et al., who showed that there is a positive correlation between systemizing abilities and sex-typical (feminine face) preferences in heterosexual neurotypical men^26^. This contrast might suggest that feminine face and feminine body are preferred differently by men and are not connected as one might predict.

Consistent with previous literature^21^, female participants preferred higher and more masculine WHR and mainly normal and high BMI for short-term relationships and normal BMI for long-term relationships with a male mate. Educational background only influenced short-term mate preference and cognitive style was not associated with preference for short-term relationships. None of the factors investigated in this study influenced long-term mate preference in women. Preference for normal and lower BMI in both male and female participants with sports backgrounds is consistent with the stereotypical attractiveness standards, suggesting that sports-related contexts, such as a societal expectation for ideal body shape, might have influenced their aesthetic preference^58^. Further investigation of mating preference in athletes provides the opportunity to understand the interplay between biological and sociocultural factors on mate preference.

Apart from the impact of cognitive style and educational background, women were different from men in mate preference over different individual and contextual factors (e.g., duration of relationship). The patterns of BMI and WHR preferences in short-term and long-term relationships were more correlated in women, but less correlated in men. These physical characteristics had independent effects on men’s mate preference. Women demonstrated more similar mate preference in short-term versus long-term relationships, specifically regarding WHR-based preference. In our study, the pattern of men’s BMI preference is more consistent than the pattern of men’s WHR preference in short-term and long-term relationships and men preferred low to normal BMIs. This could be explained by the roles of sociocultural norms and the emphasis of society and media on low weight as a marker of beauty^59^ and fashion, in a developing country like Iran. Our finding should be tested in other regions where high BMI can be considered attractive because it signals access to food^3^. More cross-cultural and cross-regional studies will shed light on sociocultural and geo-economical influences on mate preferences.

Furthermore, women’s consistent pattern of mate preference in a short-term and long-term relationship implies that either other physical characteristics are more important to women’s mate preference, or they down weigh the value of physical characteristics in mate preference^60,61^. In other words, access to resources or psychological characteristics of a mate may play a strong role for women when evaluating mate preference^62,63^. Furthermore, it also suggests short-term relationship would provide an assessment device for the women to investigate long-term prospects of their possible future mate with better genes in the short-term relationship^40^.However, as demonstrated before, men seem to have a stronger condition-dependent proclivity (i.e. short-term vs. long-term context)^35^. Given the long duration of the gestational period in humans and the huge investment of parenting, the short-term relationship may serve different purposes than long-term ones, particularly in men (e.g., the former more for pleasure and the latter more for reproduction) and they would search for different mates in short-term relationship (sexually experienced, with high sex drive, who does not search for commitment) while they search for young, physically attractive and loyal women in their long-term relationship^40^.

Several limitations of this study should be considered. First, though the first part of our results has low p-values and is robust, the regression-based results for comparing the effects of educational background, SQ, EQ, and digit ratio had marginally significant p-values that do not survive multiple comparisons correction and so these findings should be considered preliminary and interpreted cautiously. Second, to evaluate the impact of two aspects of masculinity (i.e. cognitive and physical) our participants' educational backgrounds were restricted to engineering and sports. Including broader educational backgrounds such as art, humanities, science, and medicine would provide more comprehensive insight into the interplay of education background and cognitive style and mate preference. Third, although we used some established physical attractiveness metrics (i.e. WHR and BMI), including a broader range of physical markers such as shoulder to waist ratio and facial cues might be more sensitive to address our research questions. Fourth, this study was conducted with individuals without neurodevelopmental, neurological, or psychiatric challenges. To assess the generalizability of findings, future research should explore mate preference in individuals with neurodivergent presentations in sociality such as autism spectrum or Williams syndrome. Fifth, indirect markers of early fetal hormonal exposure with controversial evidence (e.g., digit ratio, cognitive style) were used. Measuring fetal hormones directly and tracking their associations with future mate preference could be the subject of future studies. Finally, this study is cross-sectional. Future longitudinal assessments will inform the causal relationship between individual differences and mate preference.

In conclusion, we found that our participants generally followed stereotypical beauty norms. Men preferred lower WHR and lower BMI, irrespective of the timeframe of the relationship. Women preferred high WHR and low to normal BMI. Individual differences (having sports educational background and lower digit ratio only in men) could reinforce these stereotypical beauty norms for BMI preference for a short-term relationship. Empathizing tendency further reinforced stereotypical beauty norms of low WHR preference in men for a long-term relationship. Therefore, mate preference in men may be influenced by both biological (e.g., hormonal exposure in the fetal period marked by digit ratio and cognitive style) and social-experiential factors (e.g., educational background), while in women only social-experiential factors (e.g., educational background) contribute significantly. However, the role of these factors should be interpreted with caution. In a non-western country where female body figures are fully covered, socio-cultural factors could shadow the impact of biological and educational factors on WHR and BMI preferences. Finally, men showed stronger condition proclivity (regarding the timeframe of relationship) in mate preferences and women showed more consistent preference in short-term and long-term relationship.

## Methods

### Participants

We selected participants from two different educational backgrounds: sports/physical education and engineering. Participants included 178 heterosexual students (81 females/women, 97 males/men) from the University of Faculty of physical education and sport sciences and two different sports colleges in Tehran aged 17 to 45 years (mean age = 23.4 years, SD =  ± 3.7) and 153 heterosexual engineering students (64 females/women, 89 males/men) aged 18 to 31 years (mean age = 22.3 years, SD =  ± 2.4) from three different universities in Tehran. Individuals with a history of any serious neurological disorders such as seizure, brain tumor, moderate to severe traumatic brain injury and stroke, substance use disorders, major psychiatric illnesses, or receiving psychotropic medications were excluded. Three male participants from the physical education and sports sciences group and one female participant from engineering group were excluded due to missing data. Written informed consent was obtained from all participants and the research protocol was approved by the ethics committee of the Iran University of Medical Science (ethics code: IR.IUMS.REC.1399.1043).

### Measures

#### Empathizing quotient (EQ) questionnaire

The short-form of EQ is an instrument that measures components of empathy including cognitive empathy (understanding and/or predicting other’s belief, thought, emotion, action), affective empathy (to respond appropriately to other’s emotions), and mixed components. An example of an item of Short EQ is “I can easily tell if someone else wants to enter a conversation.” Each person scores two points if strongly agrees with an empathizing response to the item, and one point if slightly agrees with an empathizing response to the item^64^. This 22-item short-form questionnaire was developed by Wakabayashi et al. (Cronbach’s alpha = 0.9) from the original 40-item EQ questionnaire (English version)^31,65^. We followed standard protocol in translation and cultural adaptation of this 22-item questionnaire in Farsi^66^, which shows good reliability and validity^67^.

#### Systemizing quotient (SQ) questionnaire

SQ measures systemizing tendency, the drive to explore rules of a system and monitor input-operation-output behaviors. It was also developed by Wakabayashi et al. after removing extra items from the original version^65,68^. The short-form of SQ contains 25 items^65^. An example item is “If I were buying a car, I would want to obtain specific information about its engine capacity”. Each person scores two points if strongly agree with a systemizing response to the item, and one point if slightly agree with a systemizing response to the item^69^. The 25-item version is strongly correlated with the full-scale version and highly reliable (Cronbach’s alpha = 0.89)^65^. We followed standard protocol in translation and cultural adaptation of the 22-item questionnaire in Farsi^66^, which shows fairly good reliability and validity^67^.

#### Assessment of physical attractiveness

Body mass index (BMI) and waist to hip ratio (WHR) are potential determinants for physical attractiveness^14,15,70^. To assess individual mate preference we used a computerized, modified version of Singh's photographs^21,51^. All stimuli were 12 black-line drawings of female and male bodies. They varied in three weight patterns (underweight BMI, normal BMI, and overweight BMI) and four WHRs (0.7, 0.8, 0.9, and 1.0). The three different BMIs were scored as − 1 for “underweight BMI”, 0 for “normal BMI”, and + 1 for “overweight BMI” in the following analyses.

#### Digit ratio (2D:4D) assessment

The length between the palmar digital crease to the tip of the second and fourth finger of the right hand was measured directly by a ruler, with a degree of precision of 0.1 cm. The right hand is predicted to be a better marker for prenatal hormonal exposure^71^. The measurement was done according to previously published procedures^46,72^.

### Procedure

First, all participants were instructed to complete the short-forms of EQ and SQ. They were then asked to choose their ideal mate through photographs demonstrated on a 15-inch monitor and they could change their selection as many times as they wanted. The drawings were demonstrated on a 15-inch monitor and participants were asked to choose the most attractive, second most attractive, least attractive, and second least attractive figures, for a short-term relationship, and then again for a long-term relationship as their ideal mate. However, for the analysis for this study, only the most attractive selected figure was documented. Female participants rated male figures and male participants rated female figures.

### Analysis

Descriptive analysis was conducted to show the demographic presentation of the study. Multiple regression models were used to determine the relationships between the independent variables: i.e. sports/physical education (coded as 2) vs. engineering education (coded as 1) background group, age, digit ratio of the right hand, SQ and EQ, and dependent variables (i.e. indices of mate preference based on physical attractiveness). In all regression models, we used the following formula,

$$Y= {\beta }_{0}+{\beta }_{1}\times Group+{\beta }_{2}\times Age+{\beta }_{3}\times Digit ratio+{\beta }_{4}\times SQ+{\beta }_{5}\times EQ$$

where Y is BMI or WHR, in separate regression analyses. In all analyses, dependent and independent variables were normalized to a mean of zero and standard deviation of one (z-scored); so the reported betas are standardized beta. All analyses were conducted separately for male and female groups because they had seen different image sets of the opposite sex. Selections for a short-term and long-term relationship were also conducted separately. Due to the exploratory nature of this study, a p-value less than 0.05 was considered statistically significant. Regression analysis was implemented in Matlab using the *glmfit* function. We used Matlab version 2010b to analyze the data.

### Ethical approval

All procedures performed in studies involving human participants were under the ethical standards of the Iran University of medical sciences research committee and with the 1964 Helsinki declaration and its later amendments or comparable ethical standards.

### Informed consent

Informed consent was obtained from all individual participants included in the study.

## Data Availability

The datasets generated during and/or analyzed during the current study are available from the corresponding author on reasonable request.
